# Supplementary material for: Post-stroke fatigue: A factor associated with inability to return to work in patients <60 years—A 1-year follow-up
Source: PLoS One. 2021 Aug 4;16(8):e0255538. doi: 10.1371/journal.pone.0255538 (PMC8336834; doi:10.1371/journal.pone.0255538)
Supplement: S1 Data — (DOCX) [file pone.0255538.s002.docx]

Post Stroke Fatigue Coding Sheet

1. Case Number [Case #]
2. NIHSS Score
3. Stroke Onset to admission (# of days since stroke prior to admission to rehabilitation)
4. Age
5. Gender
   - 0 = Male
   - 1 = Female
6. Marriage Status [Marriage]
   - 0 = Married
   - 1 = Single
   - 2 = Divorced
   - 3 = Widowed
7. Education
   - 0 = Illiterate
   - 1 = Primary
   - 2 = Secondary
   - 3 = University
8. Employed
   - 0 = Yes
   - 1 = No
9. Full/Part Time
   - 0 = Full time
   - 1 = Part time
10. Paid Work
    - 0 = Yes
    - 1 = No
11. Self-employed
    - 0 = Yes
    - 1 = No
12. Attending School [In School]
    - 0 = Yes
    - 1 = No
13. Work Class [Class]
    - 0 = Yes
    - 1 = No
14. Drive prior to stroke [DS bf Stroke]
    - 0 = Yes
    - 1 = No
15. Side of Stroke
    - 0 = Left Brain
    - 1 = Right Brain
    - 2 = Bilateral
16. Location of stroke [location]
    - 0 = Cortical
    - 1 = Subcortical
    - 2 = Cerebellum
    - 3 = Brainstem
17. Type of Stroke
    - 0 = Ischemia
    - 1 = Hemorrhage
18. Neurologic deficits [Deficits]

- 0 = Hemiplegia
- 1 = Hemiparesis
- 2 = Dysarthria
- 3 = Dysphagia
- 4 = Neglect
- 5 = Ataxia
- 6 = Aphasia

1. FIM score on admission [FIM Admiss]
2. FIM score on discharge [FIM Disch.]
3. Fatigue Severity Scale [FSS-Admis, FSS-3 m, FSS-6 m, FSS-12 m]
4. Modified Rankin Disability Scale [MRDS-admis, MRDS-3 m, MRDS-6 m, MRDS-12 m]
5. Montreal Cognitive Assessment Score [MCAS-admis, MCAS-3 m, MCAS- 6 m, MCAS- 12 m]
6. Beck Depression Inventory [BDI-admis, BDI-3 m, BDI-6 m, BDI-12 m]
7. Return to work- 3 months [RTW-3m]
   - 0 = Yes
   - 1 = No
8. Return to Work – 6 months [RTW-6m]
   - 0 = Yes
   - 1 = No
9. Return to Work- 12 months [RTW-12m]
   - 0 = Yes
   - 1 = No
10. Return to Drive- 3 months [RTD-3m]
    - 0 = Yes
    - 1 = No
11. Return to Drive – 6 months [RTD-6m]
    - 0 = Yes
    - 1 = No
12. Return to Drive- 12 months [RTD-12m]
    - 0 = Yes
    - 1 = No
