# Supplementary material for: Post-stroke fatigue: A factor associated with inability to return to work in patients <60 years—A 1-year follow-up
Source: PLoS One. 2021 Aug 4;16(8):e0255538. doi: 10.1371/journal.pone.0255538 (PMC8336834; doi:10.1371/journal.pone.0255538)
Supplement: Attachment — Submitted filename: Response to Reviewer comments.docx [file pone.0255538.s003.docx]

July 5th, 2021

Dear Dr. Reppermund,

Thank you for providing the reviewers feedback to our manuscript. We have made revisions and provided responses to the reviewers’ comments below. We hope you will find the associated changes in the manuscript acceptable. Please let us know if you have any additional comments.

Thank you for taking the time to consider this manuscript for publication. We appreciate your time and look forward to your response.

Sincerely,

Nicole Rutkowski, Elham Sabri, & Dr. Yang

1. Please ensure that your manuscript meets PLOS ONE's style requirements, including those for file naming. The PLOS ONE style templates can be found at

<https://journals.plos.org/plosone/s/file?id=wjVg/PLOSOne_formatting_sample_main_body.pdf> and

[https://journals.plos.org/plosone/s/file?id=ba62/PLOSOne_formatting_sample_title_authors_affiliations.pdf](about:blank)

We have made changes to the manuscript to comply with the style requirements. Please let us know if there are any additional changes you would like us to make.

2. PLOS requires an ORCID iD for the corresponding author in Editorial Manager on papers submitted after December 6th, 2016. Please ensure that you have an ORCID iD and that it is validated in Editorial Manager. To do this, go to ‘Update my Information’ (in the upper left-hand corner of the main menu), and click on the Fetch/Validate link next to the ORCID field. This will take you to the ORCID site and allow you to create a new iD or authenticate a pre-existing iD in Editorial Manager. Please see the following video for instructions on linking an ORCID iD to your Editorial Manager account: <https://www.youtube.com/watch?v=_xcclfuvtxQ>

The ORCID ID has been added for the corresponding author.

3. Please include captions for your Supporting Information files at the end of your manuscript, and update any in-text citations to match accordingly. Please see our Supporting Information guidelines for more information: [http://journals.plos.org/plosone/s/supporting-information](about:blank).

The supporting information caption has been added.

Reviewer #1:

This is a single center study of 105 patients ages 18-60 who were employed prior to their first stroke and were followed for 12 months after to determine success at driving and return to work. Investigators evaluated the impact of fatigue using the Fatigue Severity Scale (FSS). They considered a score of greater than 4 significant fatigue. They found that individuals who were fatigued at each time point were less likely to return to work but that this did not have significant impact on driving. They also found that early fatigue was independently associated with not returning to work later.

This is an important topic. Post-stroke fatigue is more common than previously realized and a problem for individuals reintegrating into their prior home and workplace environments.

Generalizability- This is a relatively small group of patients with a single center. They are predominantly male and quite young.

Thank you for your comment, yes unfortunately the generalizability of this study is a limitation. We have noted this limitation on line 384-386. For age, only individuals under the age of 60 were recruited to capture a working age demographic.

Confounding factors and clinical significance- The authors do not account for many other potentially confounding causes- though do adjust for co-existing depression (using the Beck inventory), the patient's functional status (using the FIM, NIH stroke scale, and modified Rankin), and cognition (using the Moca). Other studies have found that when people remain fatigued chronically after stroke it is often due to other medical conditions, yet this is not evaluated and may be another reason why return to work is not possible. Age (if nearer retirement for example), and type of work may also be important. While I appreciate that fatigue was “independently associated”, a univariate analysis exploring other important factors AND including the relative weights of all important factors within their multivariable model (where they now just report the adjusted OR for fatigue) would be helpful to the reader to gauge the relative importance of each factor.

Based on your feedback we added age to both the multivariable and univariable models and found that age was not significantly associated with RTW in our sample. However, with age added in the model disability (mRS) became significant at 3 months but lost significance at 6 and 12 months line 168-172.

“Univariable logistic regression models with maximum likelihood estimates were used to assess the effect of fatigue as well as other independent variables including age, FIM, NIHSS, mRS, MoCA, and BDI at 3, 6, and 12 months for inability to return to work concurrently with dependent variables in separate models.”

We did also collect information on the type of work which we know to be relevant to return to work (i.e., physical labour jobs vs clerical or high professional jobs), unfortunately due to the high technology industry and federal public service of our city (Ottawa, Canada) the majority of our sample was from high professional or clerical jobs making it not possible to compare based on the type of job.

Thank you for your suggestion to include more information in the table, we have expanded table 2. to include each confounding factor outcomes for the univariable and multivariable analysis. However, we do agree it is a limitation that we did not collect data on other medical conditions, we have added to limitations on line 381-383.

“Additionally, fatigue may be impacted by comorbid medical conditions which were not assessed for in this study.”

The abstract could be improved by being clear that they are focused on younger individuals with first stroke who were employed prior to infarct.

Thank you for your comment. We have adjusted the abstract to reflect your feedback on line 18-19.

Within the Discussion, it is really too strong a statement to say that fatigue is the major driver for not returning to work, without some of the above analysis. It certainly is a contributing factor, but may not be the only, or even dominant one.

Thank you for bringing our attention to this. We have re-phrased to “These findings suggest that post-stroke fatigue may be an important factor for inability to return to work regardless of stroke severity, age, extent of disability, cognitive function, and depression” on line 266-268 and have included the suggested analysis.

# Reviewer #2:

The authors should clarify why 89% of their patients were in Clerical or technical or High professional positions. It is a very high percentage and it implies a sampling bias.

Thank you for your comment. We had added the following line to the manuscript: “The large sample of clerical or high professionals may be attributional to the nature of the city of Ottawa. As the capital of Canada, the work sector consists primarily of white-collar jobs in high technology and federal public service” on line 287-290. Due to Ottawa being a government city, the average income is also higher than the rest of Canada. This limitation does impact the generalizability of this study.

In the abstract, please report the p-values of the odds ratios

Thank you for your comment, we have added the p-values to the abstract on line 26-28.

I would suggest using the First people language, referring to people/patients with stroke, and not to stroke survivors.

Thank you for this very interesting comment! I believe this may be a language difference as it is common practice in North America to refer to people/patients with stroke as stroke survivors (and similarly cancer survivors).

The percentages of RWT reported in Introduction are those related only to 2 studies (4,5), despite authors reported that this rate highly varies. Probably it is better to report a range of percentages obtained comparing more studies (such as well done for fatigue post stroke)

Thank you for the suggestion, we have condensed the sentence and included a range of percentages for return to work on line 40-42.

The OBJECTIVES paragraph should be merged with the paragraph at the end of Introduction (without any subtitle). It is quite strange to find the objective in methods section.

Thank you for the suggestion, we have merged the objectives paragraph with the paragraph at the end of the introduction on line 76-84.

Is it possible to write the following sentence in a more clear manner: “For assessing the effect of fatigue on admission on return to work/drive, patients were assumed to have returned to work or drive if they had returned to work/drive at an earlier time point.”?

Yes, thank you for the feedback. We have reworded the sentence to the following on line 167-168:
“For the data analysis, survivors were presumed to have remained at work or drive at 6 or 12 months if they had indicated they had returned to work/drive at an earlier time point.” For example, if a survivor indicated at 3 months they returned to work, they were presumed to be working at 6 & 12 months in the statistical model.

I would suggest to report OR results as follows: unadjusted OR=41.6, 95% CI: 11.2-154.1, p=<0.001, I suggest to avoid to report p<.000

Thank you, we have adjusted the OR and p-value to reflect your suggestion on line 221-239 and throughout the manuscript.

When the OR was adjusted for confounders, were the confounders measured as fatigue at admission and also at the time of outcome, separately for the two analyses reported in the table?

Yes, all outcomes were reassessed at admission, 3 months, 6 months, and 12 months, with the exception of the FIM and NIHSS which was only measured at admission as per standard clinical practice.

Authors investigated the return to work, without dividing between the return to the same mansion or to the same working time (as done elsewhere, for example in “Return to Work and Quality of Life after Stroke in Italy: A Study on the Efficacy of Technologically Assisted Neurorehabilitation.”). It is a point to discuss, also because authors discussed that the time of driving could be reduced with respect to before stroke, the same could happen for return to work, despite not enough to allow this reduction.

Thank you we have clarified this in the text on line 121-124.

“In Canada when patients with stroke engage in rehabilitation, return to work planning involves the same employment they held prior to the stroke onset, therefore all the stroke patients in this study who returned to work would have returned to their previous employment on a modified capacity or full-time.”

However, you are correct that we did not assess specifically what was the reduction in working time, this has been added to the limitations on line 389-391.

“Thirdly, the standard process to return to work involves gradual return to modified hours and duties to previous employment, however this study did not collect information on the specific reduction of working hours.”

Thank you for the reference, it was very insightful and we have added it to the discussion.

Another fundamental aspect is the possibility or not to adapt the workplace/office and or the possibility to use or not special devices/aids for working. Authors should discuss also this aspect as done elsewhere (for example in “The effect of a workplace intervention program on return to work after stroke: a randomized controlled trial”)

Thank you for the reference and suggestion, we have added the following to the discussion:

“Workplace accommodations, especially work from home, may be more accessible to stroke survivors presently as many institutions have been compelled to develop infrastructure to support remote work given the global pandemic. However, a significant barrier for stroke survivors returning to work to physically demanding jobs remains [48]. Individualized return to work programs have been found to increase the likelihood three-fold of return to work among stroke survivors in South Africa by providing personalized accommodations and assessment of workplace challenges, though they require significant human resources they may be a worthwhile investment given the substantial economic burden of lost productivity among working aged stroke survivors” on lines 353-362.

The Authors suggested “to support and encourage patients in flexible return to work planning”, however this aspect is often decided by companies and regulated by government laws. Authors could provide suggestions for promoting laws favouring this approach, not just a suggestion to give to patients.

Yes, this is a very good point. With more published evidence on post stroke fatigue and its impacts on vocational rehabilitation issues, we hope to add updated recommendations in Canadian Stroke Best Practice Guidelines which help promote workplace regulations and policies. We have rephrased to the following:

“Some studies have recommended encouraging stroke survivors in flexible return to work planning, such as phased return to work and flexible working hours [41,44], yet dependent on the country these efforts may be hindered by employers due to a lack of governmental policies or laws. In Canada for example, medical professionals play an important role in advocating for workplace accommodations and duties for patients and the recommendations put forward are often respected by insurers and employers. However, in our experience greater resistance is encountered when advocating for patients with persistent fatigue” on line 344-348.

The Discussion would also benefit if authors report also some neurorehabilitation studies on how reduce the fatigue post-stroke and its effects

Thank you, we have added the following line (371-375) to the discussion:

“Neurorehabilitation programs such as functional electrical stimulation may be beneficial for return to work by improving walking, muscle weakness, and reducing fatigue through increased physical activity, however they may not be widely accessible [51]. Further research on neurorehabilitation programs, such as virtual reality and robot technology and whether enhanced motor learning and cognitive function is transferable to successful return to work is needed.”
